# Supplementary material for: MetaRibo-Seq measures translation in microbiomes
Source: Nat Commun. 2020 Jun 29;11:3268. doi: 10.1038/s41467-020-17081-z (PMC7324362; doi:10.1038/s41467-020-17081-z)
Supplement: Supplementary file 10 — Supplementary Data 7 [file 41467_2020_17081_MOESM10_ESM.zip › File2/Confidence_High_Taxonomy/171479_out.krona.html]

Javascript must be enabled to view this page.

members
magnitude
magnitudeUnassigned
count
unassigned
taxon
rank

171479\_out

4

2
superkingdom
4

1239
4
phylum

186801
2
class

order
2
186802

family
2
541000


SRS077231\_contig\_number\_contig-100\_1.2SRS148196\_contig\_number\_contig-100\_0.0
species
2
1898205

1897027

SRS019068\_contig\_number\_116369SRS105153\_contig\_number\_1
species
2
